# *Candida auris* in US Correctional Facilities

**DOI:** 10.3201/eid3013.230860

**Published:** 2024-04

**Authors:** Ian Hennessee, Kaitlin Forsberg, Jalysa Erskine, Argentina Charles, Barbara Russell, Juliana Reyes, Chantel Emery, Nickolas Valencia, Adrienne Sherman, Jason Mehr, Hannah Gallion, Brandon Halleck, Caleb Cox, Marcie Bryant, Deborah Nichols, Magdalena Medrzycki, D. Cal Ham, Liesl M. Hagan, Meghan Lyman

**Affiliations:** Centers for Disease Control and Prevention, Atlanta, Georgia, USA (I. Hennessee, K. Forsberg, M. Medrzycki, D.C. Ham, L.M. Hagan, M. Lyman);; Florida Department of Health, Tallahassee, Florida, USA (J. Erskine, A. Charles, B. Russell, J. Reyes, C. Emery, N. Valencia);; New Jersey Department of Health, Trenton, New Jersey, USA (A. Sherman, J. Mehr);; Indiana Department of Health, Indianapolis, Indiana, USA (H. Gallion, B. Halleck, C. Cox, M. Bryant);; Indiana Department of Correction, Indianapolis (D. Nichols)

**Keywords:** Candida auris, fungi, correctional facilities, prisons, correctional health, infection prevention and control, antimicrobial resistance, multidrug-resistant organism, United States

## Abstract

*Candida auris* is an emerging fungal pathogen that typically affects patients in healthcare settings. Data on *C. auris* cases in correctional facilities are limited but are needed to guide public health recommendations. We describe cases and challenges of providing care for 13 patients who were transferred to correctional facilities during January 2020–December 2022 after having a positive *C. auris* specimen. All patients had positive specimens identified while receiving inpatient care at healthcare facilities in geographic areas with high *C. auris* prevalence. Correctional facilities reported challenges managing patients and implementing prevention measures; those challenges varied by whether patients were housed in prison medical units or general population units. Although rarely reported, *C. auris* cases in persons who are incarcerated may occur, particularly in persons with known risk factors. Measures to manage cases and prevent *C. auris* spread in correctional facilities should address setting-specific challenges in healthcare and nonhealthcare correctional environments.

*Candida auris* is an emerging, frequently drug-resistant pathogen that is becoming more common in the United States (*1*). It typically affects patients in healthcare settings who have chronic illness, indwelling medical devices, and frequent or prolonged healthcare exposures (*2*,*3*).

The first known *C. auris* case in an incarcerated patient was reported to the Centers for Disease Control and Prevention (CDC) in 2020. Twelve additional cases were reported in 2022, including 8 cases from the same correctional facility. Correctional facilities in many states provide high-acuity long-term care onsite, and incarcerated persons can have frequent or prolonged stays in onsite and offsite healthcare facilities (*4*). Certain incarcerated patients may therefore be at risk for *C. auris* infection or colonization.

The multiple reports of *C. auris* in incarcerated patients raised questions from state health departments and departments of corrections about how to appropriately manage *C. auris* cases and mitigate transmission in correctional settings, especially given prior experiences with rapid spread of other infectious diseases in jails and prisons (*5*,*6*). Most, if not all, experience with *C. auris* is from noncorrectional healthcare facilities where *C. auris* cases have previously been detected (*3*), and information about transmission risk and prevention measures for *C. auris* or other novel or targeted multidrug-resistant organisms (MDROs) in correctional settings is lacking (*7*). To help fill this information gap, we describe *C. auris* cases reported in US patients who were incarcerated and summarize the challenges of managing patients and implementing prevention measures in correctional settings.

## Methods

Incarceration status is not a part of routine *C. auris* reporting, but health departments often notify CDC of cases with unusual epidemiology, such as cases in correctional facilities. We included in this report all *C. auris* cases in incarcerated patients that had previously been reported to CDC. We also conducted proactive outreach to state health departments and the Federal Bureau of Prisons to identify any additional cases that had not previously been reported to CDC.

We investigated all reported clinical and screening cases of *C. auris* among patients who were transferred or resided in to a jail, state prison, or federal prison in the United States after having a positive *C. auris* specimen during January 2020–December 2022. We used established CDC case definitions for clinical and screening cases (*8*). We collected epidemiologic information for each case and calculated basic descriptive statistics for *C. auris* case type, type of healthcare facility where the positive specimen was collected, patient age, patient health conditions at the time of transfer to a correctional facility, type of correctional facility where the patient was transferred, and type of housing arrangements. Through emails and phone calls, we provided open-ended questions to health departments, departments of corrections, and the Federal Bureau of Prisons about challenges with patient management and infection control and prevention for *C. auris* that they encountered or that were reported by correctional facilities.

## Results

### Patient and Facility Characteristics

During January 2020–December 2022, a total of 13 patients who had a positive *C. auris* specimen were transferred to or resided in a correctional facility (Table). Eleven patients had screening specimens, and the other 2 had clinical *C. auris* specimens from urine. All patients were under the custody of a state or federal prison when they had their first positive specimen. Twelve patients were receiving inpatient medical care at offsite healthcare facilities (i.e., in the community), and one patient was receiving inpatient care in an onsite prison medical unit. None of the patients were under the custody of a jail. The offsite healthcare facilities were in Texas, Florida, New Jersey, and Indiana, and all were in geographic areas where *C. auris* is commonly reported (*9*). 

**Table:** Characteristics of 13 patients who were discharged to correctional facilities after having a positive *Candida auris* specimen, January 2020–December 2022*

Characteristic	Value
Case information	
*C. auris* case type	
Screening	11 (85)
Clinical	2 (15)
Healthcare facility type where positive specimens were collected	
Acute care hospital	10 (77)
Long-term acute care hospital	3 (23)
Demographic information, medical devices, and underlying conditions at the time of transfer	
Age, y, median (range)	55 (26–66)
Sex	
M	12 (92)
F	1 (8)
Mechanical ventilation	3 (23)
Indwelling medical devices	5 (38)
Central venous catheter	2 (15)
Urinary catheter	2 (15)
Tracheostomy	2 (15)
Feeding tube	2 (15)
Underlying chronic conditions	12 (92)
Chronic kidney disease†	8 (62)
Type 2 diabetes	7 (54)
Cardiovascular disease	5 (38)
Chronic wounds	2 (15)
Correctional facility where patient was transferred	
State prison	12 (92)
Federal prison	1 (8)
Transfer location within correctional facility	
Onsite medical unit	9 (69)
Onsite medical unit, then to general population unit	1 (8)
General population unit	1 (8)
Unknown	2 (15)

*Values are no. (%) except as indicated. †Including end-stage renal disease (n = 4).

Median patient age was 55 years (range 26–66 years) (Table). Twelve of 13 patients were men. At the time of transfer, 3 patients were on mechanical ventilation, and 5 had >1 indwelling medical device, such as central venous catheters and tracheostomy tubes. All but 1 patient had chronic health conditions, including chronic kidney disease, diabetes, chronic cardiovascular disease, and chronic wounds; chronic kidney disease (n = 8) and diabetes (n = 7) were the most common chronic health conditions.

Patients were transferred from healthcare facilities to prisons in Texas, Florida, New Jersey, Indiana, and Missouri. Among 11 patients for whom detailed transfer or residence information was available, 1 was transferred directly to a general population housing unit within a prison and housed with roommates, and 10 were initially transferred to or resided in onsite medical units located within prisons. Among those initially transferred to a medical unit, 1 patient was housed in a single-patient room in the medical unit and died approximately 1 week after transfer, although it was not known whether the death was caused by *C. auris* infection. One was initially housed in a single-patient room in a medical unit for 2 months before being transferred to a general population unit and housed with roommates. The remaining 8 patients remained in onsite medical units as of last follow-up because of continuing medical needs unrelated to *C. auris* colonization. 

### Challenges Encountered and Public Health Responses for Managing *C. auris* Cases

Correctional facility staff reported several challenges in managing *C. auris* cases and implementing infection prevention and control (IPC) and response measures in prisons. Staff reported difficulties isolating patients in onsite medical units because of a lack of individual medical isolation rooms and concerns for patient well-being during prolonged isolation. Three facilities reported working with health departments to conduct colonization screening for patients at high risk with overlapping stays in the units. One additional *C. auris* case was identified through these screening efforts, although information about the scope and completeness of screening was not available. Facilities that conducted screening also noted concerns about ensuring voluntary consent for screening participation, given that incarcerated patients might not feel that refusal was an option. Some facilities also noted that nonmedical staff and incarcerated workers who perform environmental cleaning and disinfection in medical units did not have adequate training in IPC and the proper use of personal protective equipment.

Facilities also reported concerns about how to minimize risk to roommates or other close contacts of patients with *C. auris* in general population units and concerns about patients with *C. auris* experiencing stigma or threats from other incarcerated persons or staff. Facilities addressed those concerns by housing patients with roommates who did not have medical risk factors for *C. auris* colonization and providing education about *C. auris* to roommates and others assigned to housing in close proximity to the patient. Facilities reported challenges providing soap and alcohol-based hand sanitizer (ABHS) to patients and other incarcerated persons because of internal policies restricting providing those items for security reasons.

State health departments reported difficulties coordinating across multiple jurisdictions and agencies (e.g., coordinating colonization screening with the department of corrections, prison management, and contracted medical providers). One state health department reported overcoming these challenges by leveraging a preexisting relationship between the state health department and the department of corrections, which enabled rapid information sharing and decision-making between departments. Facilities also noted challenges in ensuring that patients’ *C. auris* status was consistently communicated to relevant staff within the facility and during transfers to other correctional facilities or to offsite healthcare settings.

## Discussion

This report describes cases of *C. auris* reported among patients in US correctional facilities. Cases occurred among patients in geographic areas with high *C. auris* prevalence and with recent hospital admissions, consistent with prior reports that *C. auris* is predominantly transmitted and acquired in healthcare environments (*2*,*3*). Although underlying conditions were common, not all patients had typical risk factors for *C. auris* at the time of transfer back to the correctional facility. However, data about risk factors were not collected systematically and did not necessarily reflect possible risk factors present at the time of *C. auris* acquisition. Although all but one patients in this report had their first positive *C. auris* specimens identified at offsite hospitals, transmission in onsite medical units was not fully assessed because comprehensive screening of healthcare contacts was not conducted for all cases. In the future, comprehensive screening in response to cases in correctional medical units could help determine whether transmission is occurring in these settings.

This report highlights unique challenges to *C. auris* patient management and IPC in correctional medical settings. Although no setting-specific guidance for *C. auris* response in correctional facilities exists, strategies outlined in MDRO containment guidance for healthcare settings would apply (*10*,*11*). Those guidelines focus on ensuring adherence to recommended IPC practices, conducting screening of healthcare contacts or new admissions on the basis of the local epidemiology, and communication about patients’ *C. auris* status during transfers to other facilities. Specific guidance for different facility types (e.g., nursing homes, long-term acute care hospitals, and dialysis clinics) should be adapted for correctional facility medical units on the basis of the type of unit and acuity of care provided (*10*–*12*). Strong communication and collaboration between public health agencies and correctional facilities can increase awareness about resources needed to implement IPC policies and facilitate timely response to MDROs. Facilities could work with local health departments to proactively assess their IPC practices and plan *C. auris* response activities after a case is identified (*13*). Health departments can also share local epidemiologic information to guide practices in correctional medical units, such as screening.

Risk for *C. auris* transmission in nonhealthcare settings is not completely understood, but persons in nonhealthcare settings generally lack typical risk factors for *C. auris* (*3*). No evidence exists to suggest that incarcerated patients colonized with *C. auris* should not be discharged to the facility’s general population. Disposition and decisions to discharge patients from medical units should be based on clinical criteria and healthcare need, not on *C. auris* status alone (*10*). Management for patients in general population units could draw from methicillin-resistant *Staphylococcus aureus*, tuberculosis, and other corrections-specific infectious disease guidance, which focus on routine health education and providing soap or ABHS to patients and close contacts, such as roommates (*5*,*6*,*14*–*16*). ABHS was provided in many facilities during the COVID-19 pandemic, and such policies could be adapted to ensure access to ABHS (*17*). Screening of contacts, such as those with frequent inpatient healthcare exposures, could be considered in specific situations, such as those outlined in MDRO containment guidance for residential care settings (*11*). Housing patients in general population units with low-risk roommates, such as those without underlying medical conditions or indwelling medical devices, could further reduce infection risk. Communication of MDRO status should always occur when a patient is transferred to offsite healthcare facilities or to different correctional facilities. To reduce the risk for stigma, knowledge of a patient’s MDRO status should be restricted to staff who need this information to ensure that the patient is housed and receives medical care according to recommended IPC principles.

One limitation of this report is that all cases in incarcerated patients may not have been included because *C. auris* case detection and reporting may be incomplete and incarceration status is not a part of routine case reporting. Data on patient medical conditions also were collected in a nonsystematic manner and only for those present at the time of transfer, not at the time *C. auris* was acquired; some patients may have had risk factors for *C. auris* that were not reported (*3*). Information about onsite medical units (e.g., bed counts, acuity of care, and infection control compliance) also was not systematically collected but could improve characterization of potential facility-level risk factors for *C. auris* transmission. Furthermore, assessing where transmission occurred was not possible because persons can be colonized for a prolonged period, *C. auris* screening information was not available for offsite hospitals, and comprehensive screening was not conducted in all onsite medical units.

Although uncommon, *C. auris* colonization or infection may occur in persons who are incarcerated, particularly persons with known *C. auris* risk factors and recent healthcare encounters in geographic areas with high *C. auris* prevalence. Measures to manage cases and prevent *C. auris* spread in correctional facilities should account for the unique challenges of implementing recommended IPC and response measures and caring for patients with *C. auris* in healthcare and nonhealthcare environments within correctional settings.
